# Effects of adenosine A_2A _receptor activation and alanyl-glutamine in *Clostridium difficile* toxin-induced ileitis in rabbits and cecitis in mice

**DOI:** 10.1186/1471-2334-12-13

**Published:** 2012-01-20

**Authors:** Cirle Alcantara Warren, Gina M Calabrese, Yuesheng Li, Sean W Pawlowski, Robert A Figler, Jayson Rieger, Peter B Ernst, Joel Linden, Richard L Guerrant

**Affiliations:** 1 Division of Infectious Disease and International Health, Department of Medicine, University of Virginia, Charlottesville, Virginia; 2 Division of Inflammation and Biology, La Jolla Institute for Allergy and Immunology, La Jolla, California; 3 Department of Pathology, University of California San Diego, San Diego, California; 4 Department of Molecular Physiology and Biological Physics, University of Virginia, Charlottesville, Virginia; 5 Dogwood Pharmaceuticals, Inc, Charlottesville, Virginia

## Abstract

**Background:**

Severe *Clostridium difficile *toxin-induced enteritis is characterized by exuberant intestinal tissue inflammation, epithelial disruption and diarrhea. Adenosine, through its action on the adenosine A_2A _receptor, prevents neutrophillic adhesion and oxidative burst and inhibits inflammatory cytokine production. Alanyl-glutamine enhances intestinal mucosal repair and decreases apoptosis of enterocytes. This study investigates the protection from enteritis by combination therapy with ATL 370, an adenosine A_2A _receptor agonist, and alanyl-glutamine in a rabbit and murine intestinal loop models of *C. difficile *toxin A-induced epithelial injury.

**Methods:**

Toxin A with or without alanyl-glutamine was administered intraluminally to rabbit ileal or murine cecal loops. Animals were also given either PBS or ATL 370 parenterally. Ileal tissues were examined for secretion, histopathology, apoptosis, Cxcl1/KC and IL-10.

**Results:**

ATL 370 decreased ileal secretion and histopathologic changes in loops treated with Toxin A. These effects were reversed by the A_2A _receptor antagonist, SCH 58261, in a dose-dependent manner. The combination of ATL 370 and alanyl-glutamine significantly further decreased ileal secretion, mucosal injury and apoptosis more than loops treated with either drug alone. ATL 370 and alanyl-glutamine also decreased intestinal tissue KC and IL-10.

**Conclusions:**

Combination therapy with an adenosine A_2A _receptor agonist and alanyl-glutamine is effective in reversing *C. difficile *toxin A-induced epithelial injury, inflammation, secretion and apoptosis in animals and has therapeutic potential for the management of *C. difficile *infection.

## Background

*Clostridium difficile *is the most common known cause of nosocomial diarrhea. Recently, the incidence and the frequency of morbidity and mortality from *C. difficile *infection (CDI) have increased [1,2]. These have been attributed to the BI/NAP1/III/027 strain which is fluoroquinolone resistant, carries an 18 bp deletion in the *tcdC *gene (a possible negative regulator of toxin gene production), produces binary toxin and displays hypertoxigenic properties [3]. Current treatment consists of antibiotics that suppress normal flora. While transiently effective, these treatments result in a 20-30% relapse rate, prolonged shedding and reacquisition of *C. difficile *[4]. Moreover, CDI secondary to the emerging strain has already been reported to be less responsive to metronidazole and is associated with more relapses compared with the historical strains [5]. There are now more patients requiring colectomies and ICU care. New non-antimicrobial approaches to treatment are urgently needed for CDI.

Several clinical studies have shown that glutamine and its stable derivative, alanyl-glutamine, improve gastrointestinal mucosal structure and function following injury from chemotherapy, radiotherapy or prolonged parenteral nutrition [6]. In addition to its role as an important building block for protein synthesis, glutamine also is a regulator of intracellular kinases [7], redox status [6], cell proliferation [8], apoptosis [9] and heat shock protein expression [10]. *C. difficile *toxin A induces activation of caspases 6, 8, 9 and 3 in intestinal epithelial cells [11]. Glutamine and alanylglutamine have been shown to inhibit caspase 8 and decrease toxin A-induced apoptosis [12]. Activation of adenosine A_2A _receptors attenuates inflammation in many tissues. A_2A _receptor activation in neutrophils decreases production of reactive oxygen species. In macrophages A_2A _receptor activation decreases secretion of inflammatory cytokines (e.g. IL-2, IL-12, IL-6, TNFα) and increases IL-10 [13,14]. In mouse ileal tissues, ATL 313, another adenosine A_2A _receptor agonist, reduced *C. difficile *toxin A-induced mucosal injury, neutrophillic infiltration and cell death [15].

In this study we examined the effect of combining alanyl-glutamine and ATL 370 on secretion and mucosal structural damage in *C. difficile *toxin A-induced enteritis in rabbits and cecitis in mice. We also evaluated apoptosis by immunohistologic staining in the rabbit ileum and the secretion of inflammatory (keratinocyte-derived chemokine, KC) and anti-inflammatory (IL-10) cytokines in the mouse cecum.

## Methods

### Rabbit ileal loop model

Animal protocols were approved by the University of Virginia Animal Care and Use Committee. New Zealand White rabbits weighing approximately 2 kg were fasted overnight. The rabbits were anesthetized with ketamine (60-80 mg/kg) and xylazine (5-10 mg/kg), administered intramuscularly or by inhalational isoflurane. A midline abdominal incision was made to expose the small bowel. After the ileum was flushed with 5-10 ml of PBS, 8-10 loops were created by ligation using double ties and a 1-cm interval was left between the loops. Control loops were injected with either PBS (1 ml/loop) alone or *C. difficile *toxin A (TechLab, Blacksburg VA) (10-20 μg/loop dissolved in 1 ml of PBS). Treated animals received alanyl-glutamine (Sigma-Aldrich) at a final concentration of 3, 10, 30 or 100 mM injected into the intestinal lumen just prior to toxin A injection. The ligated loops were then returned to the abdomen and the abdominal wall sutured closed. Treated animals also received either the adenosine A_2A _receptor agonist, ATL 370 (Dogwood Therapeutics), at 0, 1.5 ng, 15 ng or 150 ng dissolved in 250 uL PBS by intravenous route immediately after administration of toxin A (time 0) and 2 h after the addition of toxin A. A separate experiment was performed using equimolar concentrations (3.5 ng, 34.5 ng, or 345 ng) of adenosine A_2A _receptor antagonist, SCH 58261 (Tocris Bioscience), given intravenously 5 min after the administration of ATL 370 (at time 0 and 2 h later) to investigate the specificity of the agonist effect.

### Mouse cecal loop model

To confirm results from the rabbit experiments and determine the effects of treatment on cytokine production, mouse cecal loop experiments were also performed. C57BL/6 male mice, weighing 23-25 g each, were fasted overnight. The mice were anesthetized with ketamine (60-80 mg/kg) and xylazine (5-10 mg/kg), administered intramuscularly. A midline abdominal incision was made to expose the cecum. After flushing with PBS, a ligature was placed each at the ileal and colonic openings of the cecum, while avoiding ligation of the vascular supply. Control loops were injected with either PBS (200 μL/loop) alone or *C. difficile *toxin A (20 μg/loop dissolved in 200 μL of PBS). Treated animals received alanyl-glutamine at a final concentration of 100 mM injected into the cecal lumen just prior to toxin A injection. The ligated cecal loops were then returned to the abdomen and the abdominal wall sutured closed. Treated animals also received either the adenosine A_2A _receptor agonist, ATL 370, at a dose of 3 ng/g by subcutaneous route, immediately after administration of toxin A (time 0).

### Secretion and histopathology

After 4-5 h of incubation, the animals were euthanized and the abdomen incised. The intestines were exposed and the lengths of each ligated ileal loop were measured. Intestinal fluid was extracted from the loop and quantified. The volume:length ratio (V:L) was calculated in milliliters per centimeter per loop. The gross description (clear, serous, purulent, or bloody) of the collected fluid was also noted. One set of tissues was fixed with 10% formalin and stained with hematoxylin-eosin for histopathology. Each slide was read blindly by at least 2 investigators (CAW, SWP, GC). A grading scale for histopathology of intestinal tissues, previously formulated and published [16-18], was used to grade each slide according to mucosal disruption, cellularity, and vascular congestion. The final histopathologic score is the mean of the scores of all graders. Samples of intestinal tissue from control and treated loops were placed in liquid nitrogen for subsequent protein and RNA extraction.

### *In situ *apoptotic staining

Paraffin-embedded tissues were analyzed for apoptotic cells using ApopTag^® ^Peroxidase *In Situ *Apoptosis Detection Kit. All steps were performed according to the manufacturer's instructions. Crystal Violet free Methyl Green was used as the counterstain. Images were obtained by Olympus BX41TF with Digital Camera and MIcrosuite Pathology provided by the University of Virginia Biorepository and Tissue Research Facility. Apoptosis was quantified by counting peroxidase-stained cells in 3 randomly chosen high power fields (hpf) per tissue section quadrant. The total number of cells was then divided by 12 (3 hpfs × 4 quadrants) and the reading reported as number of apoptotic cells per hpf.

### KC, IL-10 enzyme-linked immunosorbent assays (ELISA)

Detection and quantification of KC and IL-10 proteins present in mouse cecal samples were determined by using Mouse CXCL1/KC and IL-10 Quantikine ELISA kits (R&D Systems), respectively. Cecal tissues, harvested from the mouse cecal loop model described above, were minced, bead beat for 1 min twice (with 2 min interval on ice in between bead beating) and lysed in NP-40 lysis buffer for 20 min on ice, followed by spinning at 14,000 rpm at 4^°^C for 30 min. Protein concentration in a 1:10 dilution of the supernatant was measured using bicinchoninic acid assay (BCA) following the manufacturer's instructions (Pierce Thermo-Fisher Kit) [19]. ELISA on the mouse cecal lysates was performed according to manufacturer's instructions [20,21]. Both sample sets were analyzed in triplicate. Of note, antibodies to rabbit IL-10 and IL-8 are not readily available and homologous to human sequences by only 80% and 78%, respectively. Thus, we opted to use mouse tissues to detect cytokine production.

### KC and IL-10 gene expression assay

RNA was extracted from mouse cecal tissues using Qiagen RNeasy mini Kit according to manufacturer's instructions. RNA concentration was quantified and checked for purity (A260:280 ratio) by standard spectrophotometry (Biophotometer, Eppendorf, Hamburg, Germany). Synthesis of cDNA by Reverse Transcriptase PCR was performed using SuperScript III First-Strand Synthesis System SuperMix (Invitrogen) with the use of oligo (dT) as primers. cDNA was used in quantitative PCR for measuring KC and IL-10 expression compared to hypoxanthine guanine phosphoribosyl (HPRT) expression. The primers used were: murine HPRT (forward: 5'-TCC CTG GTT AAG CAG TAC AGC CCC-3; reverse: 5'-CCA ACA AAG TCT GGC CTG TAT CCA A-3') (GenBank no. NM_013556.2), IL-10 (forward: 5'-GGC GCT GTC ATC GAT TTC TCC CC-3'; reverse: 5'-GGC CCT GTA GAC ACC TTG GTC TTGG-3') (GenBank no. NM_010548.2) and Cxcl1/KC (forward: 5'-CGC ACG TGT TGA CGC TTC CCT-3'; reverse: 5'-GTC CCG AGC GAG ACG AGA CCA-3') (GenBank no. NM_008176.3). The reaction was performed in a Bio-Rad iCycler iQ multicolor PCR Detection System using iCycler software (version 3.0). The relative gene expression was determined using the 2^-ΔΔCt ^(Livak) method using HRPT as the housekeeping gene [22].

### Statistical analysis

Continuous variables, such as V:L, histopathologic score, protein levels, were expressed as means ± standard error of the mean (SEM). For statistical comparison of the magnitude of secretion, the amount of inflammation, and protein levels, a one-way analysis of variance (ANOVA) with Bonferroni post-hoc test to compare multiple selected pairs was performed. To compare 2 groups of treatment only, a 2-tailed Student's unpaired *t *test with Welch's correction was applied.

## Results

### Adenosine A_2A _receptor agonist, ATL 370, improved *C. difficile *toxin A-induced secretion and epithelial injury in a dose-dependent manner and these effects were reversed by the receptor antagonist, SCH 58261

**Figure 1:**
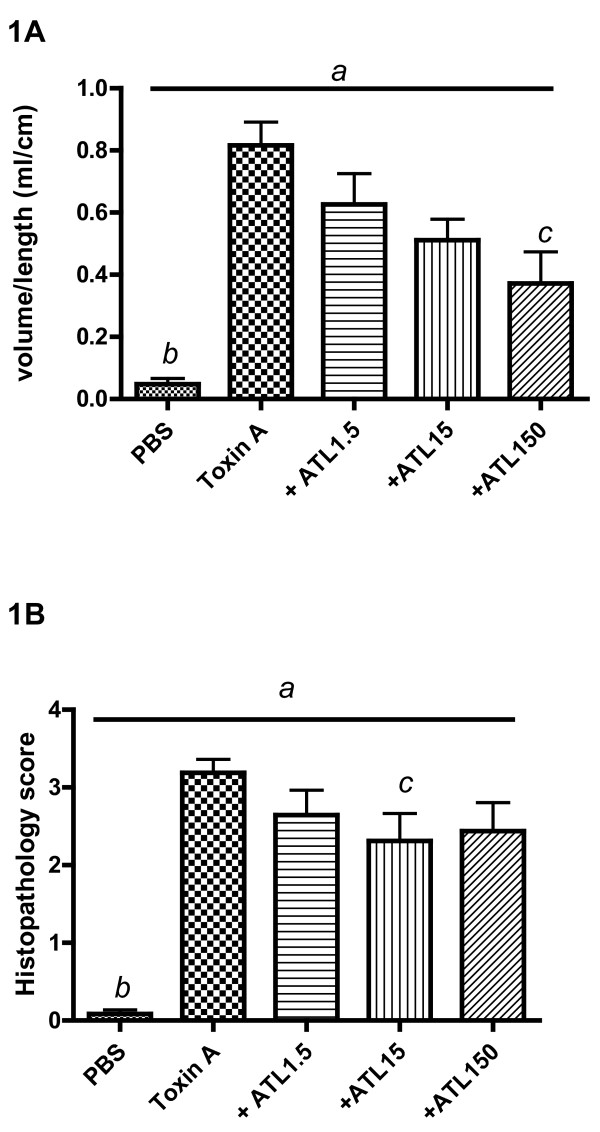
**Effects of ATL 370 alone on *C. difficile *toxin A-induced secretion and epithelial injury**. Increasing doses of intravenous ATL 370 (adenosine A_2A _receptor agonist; ng) decreased toxin A (10 μg)-induced intraluminal fluid accumulation (**A**) and histopathology scores (**B) **in rabbit ileal loops. Panel 1A: *a*- *P *< 0.0001 (one-way ANOVA), and *b*(PBS vs. Toxin A)- *P *< 0.001, *c*(Toxin A vs ATL150)- *P *< 0.01 (Bonferroni's). Panel 1B: *a*- *P *< 0.0001 (one-way ANOVA), *b*(PBS vs. Toxin A) - *P *< 0.001 (Bonferroni's), *c *(Toxin A vs ATL15)- *P *= 0.04 (Unpaired *t-test *with Welch's). Results were data pooled from 8 to 28 loops from 2 to 5 rabbits per group from 5 separate experiments.

**Figure 2:**
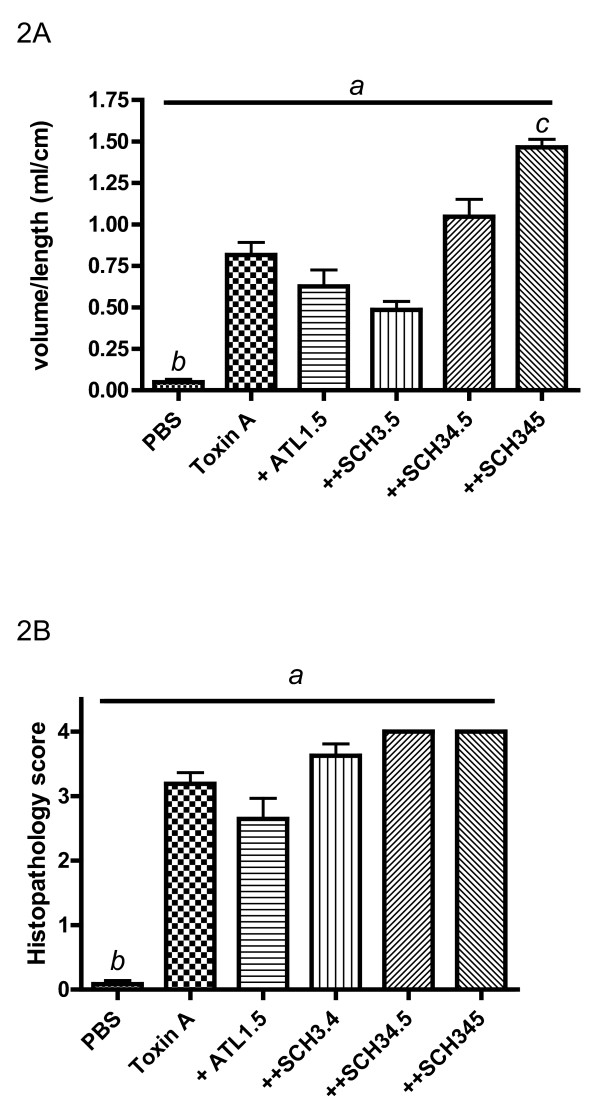
**Effects of SCH-58261 treatment on ATL 370-treated *C. difficile *toxin A-challenged loops**. Increasing doses of adenosine A_2A _receptor blocker, SCH 58261 (given IV), reversed the effects of ATL 370 (1.5 ng IV) on secretion (**A**) and epithelial injury (**B**) in a dose-dependent manner in rabbit ileal loops. 3.5 ng of SCH 58261 is equimolar to 1.5 ng of ATL370. Panel 2A: *a*- *P *< 0.0001 (one-way ANOVA), and *b*(PBS vs. Toxin A)- *P *< 0.001, *c*(Toxin A vs ATL + SCH)- *P *< 0.01 (Bonferroni's). Panel 2B: *a*- *P *< 0.0001 (one-way ANOVA), *b*(PBS vs. Toxin A)*- P *< 0.001 (Bonferroni's). Results were data pooled from 4 to 28 loops from 1 to 6 rabbits per group from 5 separate experiments.

Increasing doses of IV ATL 370 produced decreased fluid volumes in the rabbit intestinal lumen (Figure 1A). Histopathology scores did not further improve beyond ATL 370 15 ng/rabbit IV (Figure 1B). However, increasing doses of SCH 58261 yielded a dose-dependent reversal of the ATL370 effect in both secretion and histopathology score suggesting adenosine A_2A _receptor involvement in these responses (Figure 2). SCH 58261 increased secretion and histopathology score over baseline, suggesting some activation of A_2A _receptors by endogenous adenosine.

### ATL 370 and alanyl-glutamine synergistically decreased toxin A-induced ileal secretion and epithelial injury

**Figure 3:**
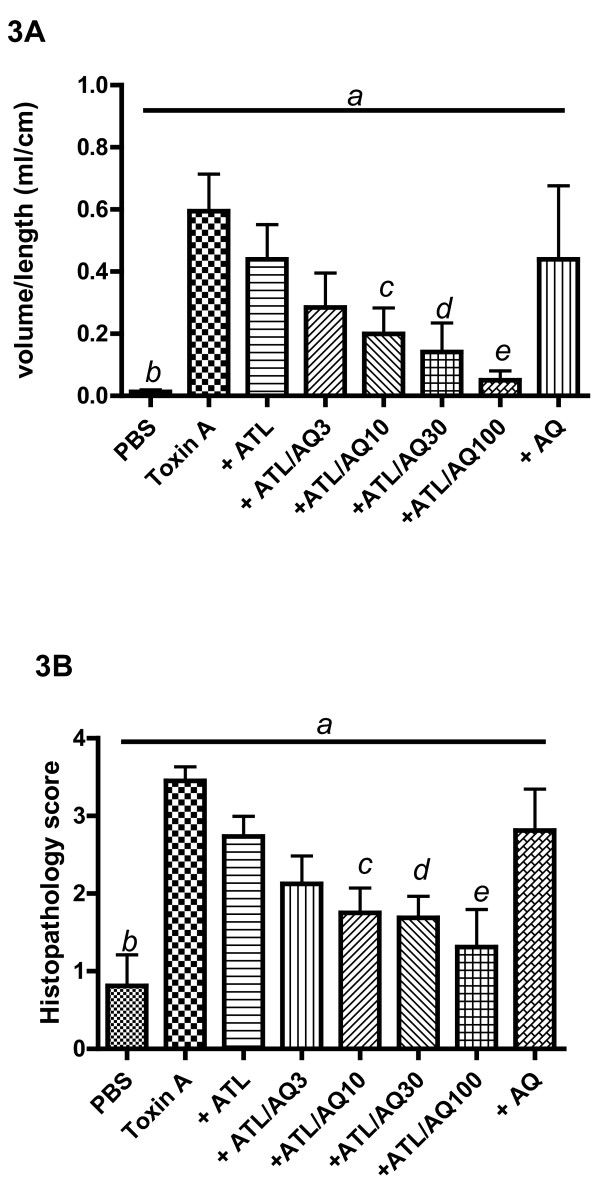
**Effects of low dose ATL 370 and alanyl-glutamine on *C. difficile *toxin A-induced secretion and epithelial injury**. Varying doses of alanyl-glutamine (AQ; mM) in the lumen when given in addition to intravenous ATL 370 (1.5 ng) showed decrease in toxin A (10 ug)-induced intraluminal fluid accumulation (**A**) and epithelial injury (**B**) in rabbit ileal loops. Panel 3A: *a*- *P *= 0.08 (one-way ANOVA), and *b *(PBS vs. Toxin A)*- P *= 0.0002, *c *(Toxin A vs ATL/AQ10)*- P *= 0.02, *d *(Toxin A vs ATL/AQ30)*- P *= 0.01, *e *(Toxin A vs ATL/AQ100)*- P *= 0.0004 (Unpaired *t-test *with Welch's). Panel 3B: *a*- *P *< 0.0001 (one-way ANOVA), and *b*(PBS vs. Toxin A)*- P *< 0.001, *c *(Toxin A vs ATL/AQ10)*- P *< 0.01, *d *(Toxin A vs ATL/AQ30)*- P *< 0.01, *e *(Toxin A vs ATL/AQ100)*- P *< 0.001 (Bonferroni's). Results were data pooled from 4 to 16 loops from 2 rabbits per group from 2 separate experiments.

**Figure 4:**
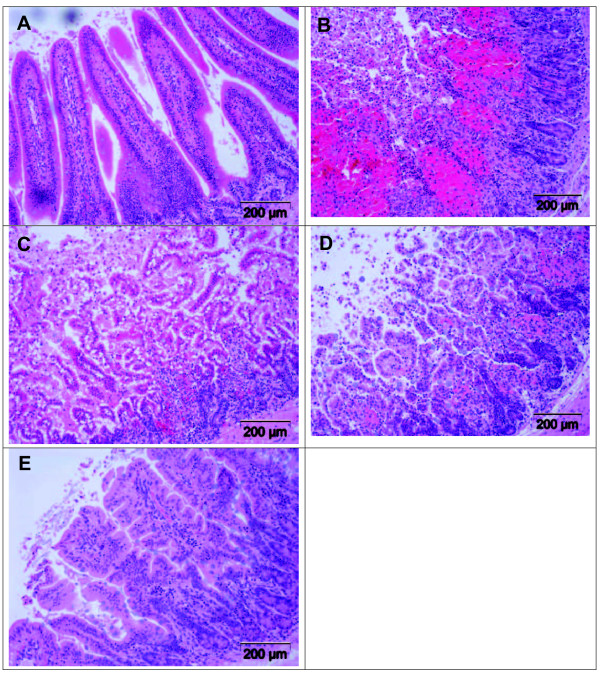
**Effects of ATL 370 and alanyl-glutamine on *C. difficile *toxin A-induced ileal histopathology**. Toxin A (10 μg, **B**) caused mucosal disruption, increased cellularity and vascular congestion compared with PBS (**A**) in rabbit ileal tissues. Treatment with intravenous ATL 370 (1.5 ng, **D**) or intraluminal alanyl-glutamine (100 mM, **C**) reduced some epithelial injury. Marked preservation of mucosal architecture was noted in tissues treated with the combination of ATL and alanyl-glutamine (**E**)

As shown in Figure 3A, rabbit intestinal loops challenged with toxin A displayed a 98% increase in hemorrhagic secretion (vol/length) compared with loops treated with PBS only. Toxin A-challenged rabbits receiving either a low IV dose of ATL 370 (1.5 ng) or intraluminal alanyl-glutamine treatment (100 mM) displayed reduced secretion, each by 27%, however, this difference did not reach statistical significance. However, in rabbits treated with both ATL 370 (1.5 ng IV) and alanyl-glutamine (10-100 mM/loop), further dose-dependent decreases in secretion were noted. The V:L decreased by 53%, 67%, 77% and 92% in loops treated with ATL 370 and alanyl-glutamine at 3, 10, 30 and 100 mM, respectively. As shown in Figure 3B, by histopathology, toxin A-stimulated intestinal tissues had significantly elevated histopathology score compared to PBS-stimulated tissues alone. ATL 370 (1.5 ng) and alanyl-glutamine (100 mM) individually decreased histopathology scores by 21% and 19%, respectively. Similar to what was observed with secretion, combination treatment resulted to dose-dependent improvement in histopathology (score improved by 38%, 49%, 51% and 62% in loops treated with ATL 370 and alanyl-glutamine at 3, 10, 30 and 100 mM, respectively). Representative images of toxin-challenged ileal tissues with or without treatment are shown in Figure 4.

### ATL 370 and alanyl-glutamine decreased KC and IL-10 in toxin A-stimulated mouse cecal tissues

**Figure 5:**
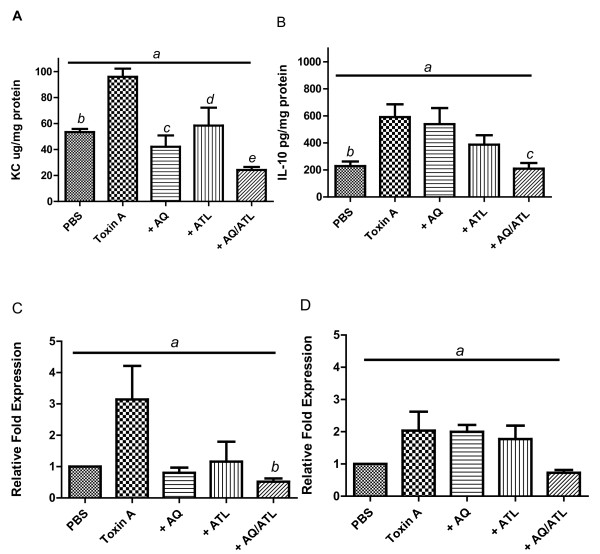
**Effects of ATL 370 and alanyl-glutamine on cytokine expression**. After four hours of incubation, alanyl-glutamine (AQ; 100 mM in loops) or ATL 370 (3 ng/g s.c.) significantly decreased *C. difficile *toxin A-induced elevation of KC secretion in mouse cecal tissues (**A**). Combination treatment with ATL 370 and AQ significantly decreased KC secretion further. Likewise, IL-10 secretion was increased by toxin A although not significantly reduced by either AQ or ATL 370 alone (**B**). Only combination treatment with ATL 370 and alanyl-glutamine significantly decreased IL-10 secretion (**B**). **C **and **D **show corresponding KC and IL-10 mRNA expressions expressed as fold-change from PBS control. Number of mice per group: Panels A&B- 5-6; Panels C&D- 3-5. Panel 5A: *a*- *P *< 0.0001 (one-way ANOVA), and *b *(Toxin A vs AQ)*- P *< 0.01, *c *(Toxin A vs AQ)*- P *< 0.001, *d *(Toxin A vs ATL)*- P *< 0.01, e (Toxin A vs ATL/AQ)*- P *< 0.001 (Bonferroni's). Panel 5B: *a*- *P *< 0.0001 (one-way ANOVA), and *b *(Toxin A vs PBS)*- P *< 0.01, *c *(Toxin A vs ATL/AQ)*- P *< 0.01 (Bonferroni's). Panel 5C: *a*- *P *= 0.04 (one-way ANOVA), and *b *(Toxin A vs ATL/AQ)*- P *< 0.05 (Bonferroni's). Panel 5D: *a*- *P *= 0.056 (one-way ANOVA)

In mouse ligated cecal tissues, KC levels were increased by 81% in toxin A-treated loops compared with PBS-treated loops (96 ± 6 vs 53 ± 2 μg/mg, *p *< 0.0001). Treatment with either AQ or ATL 370 decreased KC in intoxicated cecal tissues by 73% from the toxin A-treated loops level (Figure 5A). IL-10 secretion was more than 2-fold increased in toxin A-treated loops compared to PBS alone (591 ± 94 vs 228 ± 34 pg/mg tissue, *P *< 0.01) (Figure 5B). Alanyl-glutamine or ATL 370 alone did not reduce IL-10 secretion but when combined together caused a 65% reduction of the cytokine level (p < 0.01). KC and IL-10 mRNA expressions followed a similar trend as the ELISAs (Figure 5C and 5D, respectively).

### ATL 370 and alanyl-glutamine decreased toxin A-induced apoptosis in the intestinal tissues

**Figure 6:**
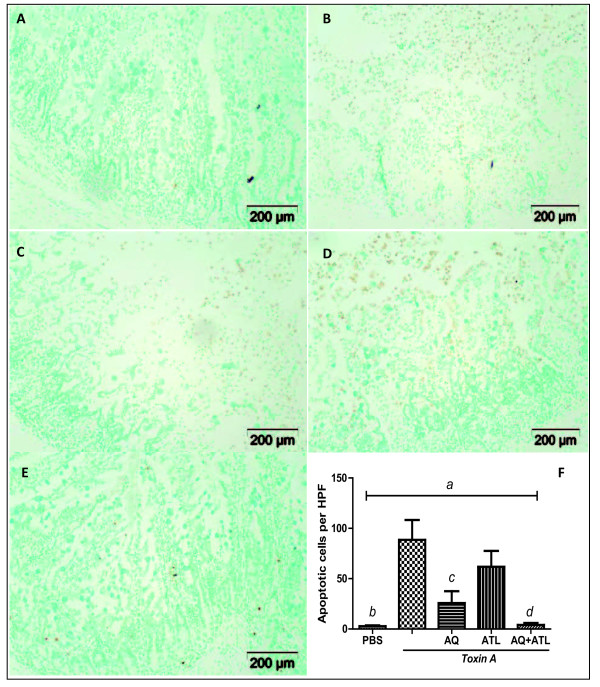
**Effects of ATL 370 and alanyl-glutamine on *C. difficile *toxin A-induced apoptosis**. Toxin A (10 μg, **B**) caused significant apoptosis in the rabbit ileal tissues compared with PBS (**A**). Treatment with ATL 370 (1.5 ng/rabbit IV, **D**) or alanyl-glutamine (AQ;100 mM in loops, **C**) caused some reduction of apoptotic cells but only significant difference was noted with treatment with AQ. A marked decrease in apoptotic cells was noted in tissues treated with the combination of ATL and alanyl-glutamine (**E**). *a*- *P *< 0.0001 (one-way ANOVA), and *b *(PBS vs. Toxin A)*- P *< 0.001, *c *(Toxin A vs ATL/AQ10)*- P *< 0.01, *d *(Toxin A vs ATL/AQ100)*- P *< 0.001 (Bonferroni's). Apoptotic cells were counted in 12 high power fields (3 hpfs/quadrant) per slide from 3 to 4 ileal specimens per group.

Toxin A treated rabbit intestinal tissues (Figure 6A) showed marked apoptotic activity compared with PBS control tissues (Figure 6B). Due to mucosal sloughing, most of the apoptotic cells were located in the lumen of toxin A-treated tissues. Treatment with alanyl-glutamine (Figure 6C) or ATL 370 (Figure 6D) alone resulted in increased mucosal preservation. Apoptotic cells were noted both in the lumen and within the intact mucosa and submucosa. Alanyl-glutamine treatment exhibited better anti-apoptotic activity than ATL 370. Combination of alanyl-glutamine and ATL 370 resulted in further decrease in the number of apoptotic cells and increased preservation of epithelial architecture (Figure 6E).

## Discussion

*C. difficile *toxin A has repeatedly been shown in numerous *in vitro *and *in vivo *studies to elicit an exuberant inflammatory response causing recruitment of inflammatory cells (neutrophils, macrophages and mast cells), secretion of inflammatory cytokines (IL-8, IL1B, TNFα), cell death, mucosal damage and intestinal secretion [23-25]. Although both *C. difficile *toxins A and B glucosylate Rho GTPases [25] and are important in pathogenesis during infection [26,27], most animal models of intoxication--rabbits, mouse, rats and hamsters, are more responsive to the enterotoxic effects of toxin A than toxin B [23,28]. This study has shown that adenosine A_2A _receptor agonist, ATL 370, ameliorates *C. difficile *toxin A-induced intestinal epithelial disruption, inflammation and secretion in the ligated loop model of enteritis in rabbits. These effects were augmented by co-administration of the dipeptide, alanyl-glutamine. Significant decreases in IL-8 and IL-10 in mouse cecal tissues were also mostly noted with the co-administration of ATL370 and alanyl-glutamine.

Adenosine is a naturally occurring purine nucleoside that is produced in many tissues in response to inflammatory or hypoxic stimuli. There are 4 known G-protein coupled adenosine receptors- A_1_, A_2A_, A_2B _and A_3_. Adenosine produces anti-inflammatory activity via A_2A _receptors found in immune cells including neutrophils, macrophages, dendritic cells and platelets. In the presence of tissue injury or hypoxia, generation of adenosine is increased and adenosine receptors are upregulated [29]. Activation of the A_2A _receptor with adenosine or a specific A_2A _receptor agonists inhibits recruitment of inflammatory cells and expression of inflammatory cytokines [30]. Adenosine inhibits expression of adhesion molecules (E-selectin and vascular cell adhesion molecule-1, VCAM-1) and secretion of IL-6 and IL-8 in human endothelial cells [31]. Indeed, it has previously been reported that intraluminal administration of A_2A _agonist, ATL 313, in *C. difficile *toxin A-challenged murine ileal loops decreased neutrophil infiltration as measured by myeloperoxidase activity and tissue TNFα secretion [15]. *C. difficile *toxin A-induced intestinal mucosal injury, intraluminal secretion and apoptosis were all improved in ileal tissues treated with ATL 313. Our study using two different animal models (rabbit ileal and murine cecal loops) and a different adenosine A_2A _receptor agonist (ATL 370) corroborated these previous findings. Interestingly, the high doses of ATL 370 (150 ng) used in this study did not offer additional improvement in histopathologic scores indicating that lower doses may be sufficient for an optimal effect. The observed effects of ATL 370 on intestinal secretion were dose-dependent and reversed by highly specific A_2A _antagonist, SCH 58261, also in a dose-dependent manner suggesting the specificity of the observations to the adenosine A_2A _receptor. In addition, our study showed that the anti-inflammatory, anti-secretory and anti-apoptotic effects from treatment with A_2A _agonist may be enhanced by the addition of alanyl-glutamine.

The exact mechanism as to how glutamine and alanyl-glutamine (a highly soluble and stable derivative of glutamine) protect enterocytes from injury and modulate inflammatory response is unclear. Alanyl-glutamine is hydrolyzed to alanine and glutamine in the gut. Glutamine is the major oxidative energy source for enterocytes, being the building block for purine and pyrimidine nucleotide synthesis and precursor of glutathione in the gut [32]. Under pathologic or catabolically stressful conditions, i.e. infection or injury, glutamine is observed to be depleted and thus, it is considered as a conditionally essential amino acid [33]. Moreover, glutamine deprivation has been known to induce apoptosis in intestinal epithelial cells [34]. The protective effect of glutamine against mucosal injury and inflammation is thought to be partly mediated by heat shock proteins [10,35]. More recently, glutamine has also been considered to be an agonist of peroxisome proliferator-activated receptor gamma, which belongs to the family of nuclear receptor proteins which regulates expression of inflammatory cytokines [36]. It has also been previously shown that *C. difficile *toxin A induces apoptosis by activation of caspases 3, 6, 8, and 9 and Bid, mitochondrial damage and release of cytochrome c in T84 cells [11]. Glutamine and alanyl-glutamine inhibited activation of caspase 8 and reduced apoptosis in T84 cells and improved intestinal secretion and mucosal disruption in rabbit ileal loops challenged with *C. difficile *toxin A [12]. These findings are consistent with what we have observed where the presence of alanyl-glutamine enhanced reduction of apoptosis, inflammation, secretion and epithelial injury in ATL 370-treated, *C. difficile *toxin A-stimulated ileal tissues.

The beneficial effect of combination therapy for toxin A-induced enteritis is less explored in the literature. In this study we attempted to control both epithelial disruption and inflammatory response with an adenosine A_2A _receptor, ATL 370, and alanyl-glutamine. ATL 370 was delivered parenterally and glutamine intraluminally. Given the short incubation time (4-5 h) of the experiments, it is conceivable that systemically administered A_2A _agonist may have acted mostly on the immune cells, specifically neutrophils, and endothelial cells, which are known to express more of the A_2A _receptors than on the enterocytes, which express more of A_2B _receptors [37-39]. Indeed, other A_2A _agonists have been shown to decrease inflammatory responses and improved survival in mouse models of endotoxemia and sepsis [40,41]. Several experimental studies have shown that alanyl-glutamine is at least as effective as glutamine in improving electrolyte absorption and preserving mucosal integrity against enterotoxin or chemical insult [42-46]. Alanyl-glutamine enters enterocytes through PepT1 proton-coupled peptide transporters and release L-glutamine by the action of cytosolic peptidases [47,48]. Although glutamine may affect leukocyte-dependent inflammatory events, such as migration and release of inflammatory cytokines, in this particular study, glutamine may have caused more of the protective effect on mucosal architecture and prevention of enterocyte apoptosis than prevented recruitment of inflammatory cells. However, release of cytokines from disrupted epithelial cells contributes to recruitment of immune cells and inflammatory mediators from the later also contribute significantly to mucosal injury [49]. These joint epithelial and immune responses to a biologic insult such as an enterotoxin, possibly explains the additive amelioration of mucosal injury, secretion, and apoptosis or even the synergistic effects on the expression of cytokines with combined treatment in this study. Whether these observations will hold true in the mouse model of *C. difficile *infection remain to be seen but these preliminary findings raise the potential use of combination therapy with enteral alanyl-glutamine and systemic low-dose A_2A _adenosine agonist in the management of *C. difficile*-induced diarrhea.

## Conclusions

In conclusion, activation of A_2A _adenosine in combination with alanyl-glutamine reduced toxin-induced intestinal secretion, epithelial disruption and inflammation in the ileal loop model of *C. difficile *enteritis. A_2A _adenosine agonists and alanyl-glutamine may have therapeutic potential for the management of *C. difficile *infection.

## Competing interests

CAW, GMC, YL and SWP have no competing interests. RAF is partially funded by Dogwood Pharmaceuticals, Inc. (formerly ATL, LLC). JR is employed by Dogwood Pharmaceuticals, Inc. PBE has collaboration on NIH grant with Dogwood Pharmaceuticals, Inc. JL is a consultant for Dogwood Pharmaceuticals, Inc. RLG co-founded AlGlutamine, LLC.

## Authors' contributions

CAW conceptualized study design, performed animal experiments, collated and analyzed data, drafted manuscript. GMC participated in animal experiments, assisted in study design, performed protein and gene expression assays and immunohistochemistry. YL participated in animal experiments and assisted in mouse study design. SWP participated in rabbit experiments. RAF participated in reviewing data and manuscript. JR participated in reviewing data and manuscript. PBE critically reviewed data and manuscript. JML critically reviewed data and manuscript. RLG critically reviewed data and manuscript. All authors read and approved the final manuscript.

## Pre-publication history

The pre-publication history for this paper can be accessed here:

http://www.biomedcentral.com/1471-2334/12/13/prepub
